# Dosage of pain rehabilitation programs: a qualitative study from patient and professionals’ perspectives

**DOI:** 10.1186/s12891-018-2125-4

**Published:** 2018-06-30

**Authors:** Michiel F. Reneman, Franka P. C. Waterschoot, Elseline Bennen, Henrica R. Schiphorst Preuper, Pieter U. Dijkstra, Jan H. B. Geertzen

**Affiliations:** 1 Department of Rehabilitation Medicine, Center for Rehabilitation, University Medical Center Groningen, University of Groningen, P.O. Box 30.002, 9750 RA Haren, The Netherlands; 2 Center for Human Movement Sciences, University Medical Center Groningen, University of Groningen, Groningen, The Netherlands; 3 Department of Oral and Maxillofacial Surgery, University Medical Centre Groningen, University of Groningen, Groningen, The Netherlands

**Keywords:** Pain rehabilitation programs, Dosage, Qualitative study, Chronic pain

## Abstract

**Background:**

There is a large and unexplained practice variation in prescribed dosages of pain rehabilitation programs (PRP), and evidence regarding the optimum dosage is unknown.

**Methods:**

To explore perspectives of patients and rehabilitation professionals regarding dosages of PRP an explorative qualitative research design was performed with thematic analysis. Patients and rehabilitation professionals were recruited from three rehabilitaton centers in the Netherlands. A purposive sample of patients who completed a PRP, with a range of personal and clinical characteristics was included. Rehabilitation professionals from all different disciplines, working within multidisciplinary PRP for a minimum of two years, for at least 0.5 fte were included. Individual semi-structured interviews were conducted with 12 patients undergoing PRP, and three focus groups were formed with a total of 17 rehabilitation professionals involved in PRP.

**Results:**

All patients were satisfied with received dosage. Factors important in relation to dosage of PRP were categorized into patient related characteristics (case complexity from a biopsychosocial perspective) to treatment related characteristics (logistics and format of the program, interaction between patients and professionals), and external factors (support from others, costs, traveling distance and injury compensation). Professionals concluded that dosage was currently based on historical grounds and clinical expertise.

**Conclusion:**

Patients and professionals from different centers considered the same factors related to dosage of PRP, but these considerations (from patients and professionals) led to different dose choices between centers. PRP dosage appeared to be mainly based on historical grounds and clinical expertise. The insights of this study could assist in future research regarding optimum dosage of PRP and rehabilitation programs in general.

**Electronic supplementary material:**

The online version of this article (10.1186/s12891-018-2125-4) contains supplementary material, which is available to authorized users.

## Background

Multidisciplinary Pain Rehabilitation Programs (PRPs) are recommended to treat patients with chronic musculoskeletal pain (CMP) [1, 2]. These PRPs, based on the biopsychosocial model, aim to decrease disability and optimize participation of patients with CMP. A systematic review showed that PRPs have a moderate, but consistent, positive effect on disability and pain, compared to usual care or physical treatment programs for patients with chronic low back pain [3]. PRPs result in better self-management of pain and disability, and a reduction in healthcare utilization in treated patients was found, which may contribute to a decrease of direct and indirect costs over the long term [4].

Although effective, PRPs are relatively expensive. The multidisciplinary characteristics, as well as the high number of contact hours and total duration of PRP, provide relatively high direct costs and travel expenses for the patients. Dosage of PRP includes the total duration, the total number of contact hours, and intensity of treatment (number of contact hours per week). Differences in the dosage of PRP may lead to differences in direct and indirect costs. Choices for dosage and how dosage is established for an individual patient is unknown. A recent systematic review showed that dosage of PRP has never been studied as a primary aim and the optimum dosage of PRPs is currently unknown [5]. The studies included in that review differed in terms of dosage (total duration, from weeks to months, and regarding contact hours, from fewer than 10 to over 100 h) and effect.

Better understanding of variables that are taken into account when deciding for a particular dosage could lead to better and more efficient patient care, which will benefit patients, rehabilitation facilities, insurers and employers. This study aimed at exploring perspectives of patients and rehabilitation professionals to acquire insight into factors that may contribute to dose choices of multidisciplinary outpatient PRP. In addition, this study aimed at examining the argumentations underlying choices in dosage of PRPs in three rehabilitation centers in the Netherlands.

## Methods

A study using an exploratory qualitative design with thematic analyses was conducted to gain in-depth information about the perspectives of patients and rehabilitation professionals involved in PRPs regarding the dosage of these programs. The study was performed in three rehabilitation centers in the Netherlands. These centers were selected because of their differences in dosage, while patient characteristics of these centers are similar [6]. Individual semi-structured interviews with patients who had completed a multidisciplinary PRP, as well as focus groups with professionals working in multidisciplinary PRP, were performed. The medical ethics committee of the University Medical Center of Groningen (UMCG) granted a waiver for this study. All participants signed informed consent for participation, digital recording and the use of data.

### Participants

A purposive sample of patients was included from three rehabilitation centers in the Netherlands with the task to develop, innovate and evaluate PRPs: Adelante in Hoensbroek (RC1), located in the south; Roessingh in Enschede (RC2), located in the east and the Center of Rehabilitation of the UMCG (RC3), located in the north. The sampling strategy aimed to include patients who completed a PRP, with a range of personal and clinical characteristics: age, disability status, gender, working status, and dosage received. PRP should have been completed for at least two weeks. The second author contacted potential participants (recruited by the contact person of the rehabilitation center) via telephone to check their willingness to participate in the study and to plan the interview. They received written information about the purpose of the interview.

The contact person of the centers selected rehabilitation professionals from the rehabilitation centers. They participated in the focus groups, provided that they were working within the area of multidisciplinary PRP for a minimum of two years, for at least 0.5 fte. We aimed to include rehabilitation professionals from different disciplines involved in PRPs (i.e. occupational therapist/physiotherapist/psychologist/physiatrist). These rehabilitation professionals should be involved in determining the individual dosages of the program for the patient.

### Data collection

Data were collected between May and September 2014. A total sample of 15 patients was planned for the interviews, five patients from each rehabilitation center, with the expectation of reaching data saturation. A semi-structured interview scheme was constructed Additional file 1 for patients using open questions enabling reflection about experiences and perceptions on dosage of treatment received. The interview started with questions about the total duration and intensity of PRP, followed by questions regarding personal experiences and perceptions of treatment dosage, such as: “what did you like and dislike about the duration and intensity of the program” and “which factors, in your opinion, influence dosage of PRP?”. Also patients’ opinions regarding the relationship of dosage to return to work and personal and general costs were asked. Face-to-face interviews were performed at the rehabilitation center where the patient was treated. The interviews were scheduled for 60 min and were audio recorded. Prior to this study, an interview was pilot tested, which led to refinements in the setup of the interview and questioning technique.

For the focus group meetings, a minimum of five rehabilitation professionals of different disciplines per center were invited in order to achieve variation in perspectives on PRP dosage per rehabilitation center. Meetings were planned for 90 to 120 min and were audio recorded. An interview scheme was constructed, aiming to gain insight into the line of reasoning underlying dosage choices, as well as rehabilitation professionals’ perspectives on optimum dosage of PRPs.

All patient interviews and focus group meetings in Adelante and Roessingh rehabilitation centers were led by one author (FW), who has over 10 years of experience as an occupational therapist in pain rehabilitation. Because she was closely acquainted with the rehabilitation professionals at UMCG, a psychologist with experience in chronic pain treatment but who was not involved in the treatment of the patients led this focus group. Another author (EB) assisted in all focus groups.

### Data analysis

Interviews and focus groups were audio recorded and transcribed verbatim by two authors (FW and EB). Transcripts were imported into ATLAS.ti, version 5.2, to analyze data. Thematic analysis was considered as the appropriate approach for identifying, analyzing and reporting factors related to dosage of PRP [7]. Because of the lack of evidence regarding dosage of rehabilitation programs, especially for PRP aimed at behavior change [5], it was not possible to provide a detailed theoretical framework. Initially, both authors conducted the inductive coding of the interviews and a third researcher independently validated the codes. All codes and quotations were re-analyzed, merged, and renamed.Data saturation was assumed when no new themes emerged from a consecutive interview or focus group.

## Results

### Participants

In total, 13 patients were interviewed and 12 interviews were analyzed. Initially, five patients per center were planned, two dropped out because of logistics problems and one was excluded because the audio was impossible to transcribe. Seventeen rehabilitation professionals participated in the focus groups (Adelante in Hoensbroek: *n* = 5, Roessingh in Enschede: *n* = 5, Center for Rehabilitation of the UMCG in Groningen: *n* = 7). Different disciplines participated: occupational therapists (*n* = 4), physiotherapists (*n* = 3), movement teacher (*n* = 1), psychologists (*n* = 4), social workers (*n* = 2) and physiatrists (*n* = 3). The characteristics of participants are described in Table 1. Data saturation was reached for patient interviews and focus groups for rehabilitation professionals.

**Table 1:** Characteristics of participants and PRP dosage

	RC1	RC2	RC3
Patients	(*n* = 4)	(*n* = 4)	(*n* = 4)
Age *mean (SD)*	42 (16)	37 (11)	53 (7)
Female *%*	75	100	100
Duration of pain *n*			
1 to 2 years	0	1	2
2 to 5 years	2	0	0
> 5 years	2	3	2
Initial PDI score *mean (SD)*	27	43.3 (14.2)	31.5 (16.4)
*Missing n*	3	0	0
Rehabilitation professionals	(*n* = 5)	(*n* = 5)	(*n* = 7)
Age *mean (SD)*	41 (6)	43 (9)	39 (6)
Female *%*	60	80	71
Years of experience in PRP *mean (SD)*	7.6 (4.7)	9.0 (5.6)	7.4 (5.5)
PRP			
Range in total duration (wks)			
*Assessment*	3	1 to 2	1 to 2
*Treatment*	12	3 to 36	8 to 20
Range in contact hours			
*Assessment*	30	2	3
*Treatment*	120	10 to 150	30 to 70

*PDI* Pain Disability index, *PRP* Pain Rehabilitation Program, *RC1* Adelante in Hoensbroek, *RC2* Roessingh in Enschede, *RC3* Center for Rehabilitation of the UMCG in Groningen

### Factors related to dosage

Different codes related to dosage of PRP were derived from the interviews and focus groups. A total of 36 codes were categorized as patient-related factors (*n* = 11), treatment-related factors (*n* = 17) or external factors (*n* = 9). In addition, the codes were arranged as “shared” (indicating that they were mentioned by patients and rehabilitation professionals), “patients” (factors mentioned by patients only), and “rehabilitation professionals” (factors mentioned by rehabilitation professionals only) (Table 2).

**Table 2:** Overview of codes per category

	Factors mentioned by patients only	Shared factors mentioned by patients and professionals	Factors mentioned by rehabilitation professionals only
Patient related factors	Assertiveness	Case complexity	–
	Relapse	General status	
		Insecurity	
		Self-knowledge	
		Motivation and ability to change behavior	
		Expectancies	
		Focus on yourself	
		Acceptance of pain	
		Self-management	
		Apply lessons learned into practice	
Team & treatment related factors	Having time and opportunity to explore	Waiting time	–
	Saturation	Clarity about dosage and time plan	Lack of evidence
	Expertise of the team	Individually tailoring	Prediction of dosage of PRP
		Shared decision making	Timing to finish PRP
		Contact with rehabilitation professionals	
		Interdisciplinary functioning	
		Content of treatment	
		Treatment goals	
		Format of treatment	
External factors	Support from environment	Direct costs	Travelling time
		Indirect costs	Format of treatment
		Investment for the future	Injury compensation
		Personal and work factors	Test results or other treatments

### Patient-related factors

#### Shared factors

Both patients and rehabilitation professionals mentioned ***case complexity*** of a patient as an important factor related to dosage of PRP as well as an important issue to take into account for indication of dosage of PRP. Both patients and rehabilitation professionals described characteristics that affect dosage of PRP, such as the ***general status*** (the level of physical condition or the degree of experienced disability), as well as the degree of ***insecurity***, ***self-understanding***, ***motivation and ability to change behavior,*** in addition to the ***expectancies*** patients have regarding treatment and treatment results. (Patient 3 (P3)): *“It depends on what is going on with someone. Is he traumatized? Does he want to talk about it? Is he able to manage his problems? Does he have some self-understanding; does he know what his problem is?”*

Both patients and rehabilitation professionals mentioned that PRP requires ***focusing on yourself***. Patients and rehabilitation professionals involved with both high and low PRP dosages mentioned this focus as being related to dosage. A patient (P7) who underwent semi-inpatient PRP described: *“The inpatient format of treatment was a good experience. It was quite intense, but actually very good because you really have to focus on yourself. If I would have been at home, I would have been distracted by all the things you have to do at home*”. Focusing on oneself and on the treatment was also mentioned by a patient (P9) as motivation for preferring twice a week PRP instead of once a week: *“Yes, two times a week, then you are really engaged with the treatment. Treatment only once a week…. well, then a whole week is in between…”.* Rehabilitation professionals mentioned that a patient being able or motivated to focus on oneself results in being motivated to follow the prescribed dosage of PRP. In some cases, for example (Therapist 13 (T13)) *“if a patient is overloaded”*, being able to focus on oneself may be an important factor to consider while determining dosage.

***Acceptance of pain*** is related to the content of PRP. Participants mentioned that PRP contributed to the acceptance of pain and that patients need time to go through a process of acceptance (P4): “*I think the minimum duration of PRP should be 12 weeks. Especially because you experience a process of change in which you need time… time to make a start, because after those 12 weeks you still need to go on for yourself”.*

For patients able to ***self-manage*** pain and disabilities, participants suggested a lower dosage of PRP, as compared to an unable patient. A rehabilitation professional in a focus group (T16) described: *“Sometimes the minimum dosage of PRP is just one hour of intake. There are patients who just need some explanation to self manage their lives again. Being able to self manage pain is related to patient characteristics and behavior, the way people are, and whether they are able to cope with their pain and disabilities themselves”.*

To **a*pply lessons learned into practice,*** patients need time. (P9) *“It is a process which requires time, 6 or 8 weeks… well, that’s quite short…. You need to be aware of things, you have to practice, adjust and adopt”.* Needing time to apply lessons learned was mentioned with regard to intensity (contact hours per week) and the reduction of intensity of contact hours during PRP. (T13): *“At the end of the program, reducing the intensity of the program (fewer days or fewer contact hours per week), could support the process of putting the lessons learned into practice”.*

#### Factors from patients only

***Assertiveness*** was mentioned by patients with regard to the ability to stand up for themselves. This code is part of the goals and content of PRP and therefore patients mentioned this in relation to needing a lower dose when patients are already able to stand up for themselves.. (P8) *“I had to learn to set boundaries. My day was fully planned and I used to do whatever I was told, which resulted in being exhausted at the end of the day”.* Some patients reported that they needed support during times of ***relapse***, which may contribute to a decision to extend the program***.*** (P2): *“Everything was going well; I also felt it that way. However, I did not have the feeling that I was ready. I was afraid that I would relapse and that was why I needed some sessions”.*

#### Factors from rehabilitation professionals only

No other patient-related factors were reported by rehabilitation professionals only.

### Treatment-related factors

#### Shared factors

***Waiting time*** before and during treatment can influence, in a negative or positive way, the general status of a patient at the start of treatment, therefore having an influence on the dose of PRP. One patient (P1) described how she was able to turn the negative result of the waiting time into a positive action: *“I had to wait for a long time before start of PRP. For me personally, it has led to an increase of pain and I became depressed. Consequently, I realized I had to do something and I started doing things on my own”.* Most patients knew the total duration of PRP beforehand. Patients were satisfied with this ***clarity*** about dosage and time plan for PRP: (P4) *“I compare it with knowing when to go on holidays: the last week at work you are really into holidays. With treatment, it is the same – you have a set goal to work on in time”.* Nevertheless, besides structured dosages providing this clarity, participants emphasized that the possibility of **individually tailoring** dosage to their needs is important. The individually tailored dosage is also related to **shared decision-making.** Patients and rehabilitation professionals attached value to shared decision-making in terms of PRP dosage. Good **contact between patient and rehabilitation professionals** supported treatment and therefore could reduce dosage: (P11): *“You need to have a connection with your rehabilitation professionals; otherwise you will not make any progress”.* However, too many changes in rehabilitation professionals during treatment can impede the connection, and therefore the process, because: *“you have to tell your story again and again”*. Related to this code, patients and rehabilitation professionals mentioned that good **interdisciplinary functioning** of the team supported efficiency and could reduce PRP dosage. (P9): *“I experienced that everyone knew who I was and what I needed. Everyone had read all reports and you did not need to tell the same stories”.* Both patients and rehabilitation professionals made it clear that dosage of PRP was strongly related to the **content of treatment, treatment goals** and the **format of treatment** (e.g. inpatient versus outpatient).

#### Factors from patients only

Most patients had the **experience of having time and opportunity** during PRP to explore themselves and reach their goals. However, some patients also experienced this as a problem when the frequency was reduced from five days to two days per week. *“Content became more intensive, everything was put together in two days and everything had to go faster…I had to hurry….”.* Some patients denoted that towards the end of treatment, **saturation** of treatment content occurred. Therefore, they suggested that retrospectively, the dosage could have been reduced. (P2): *“At a certain moment, you are done with it”.* Patients stated that they trust the **expertise of the team,** both with regard to the content of treatment as well as a determination of dosage and when decisions are made regarding the need to prolong or reduce PRP dosage. (P1): *“I thought, well if that is the success of the treatment, then I am going to do it that way”*.

#### Factors from rehabilitation professionals only

The focus groups revealed that there was a **lack of published evidence** for the delivered doses of PRP. (T15) *“These programs have been developed and adapted over the years based on skills acquired by the team, and developments such as group based treatments. This provides certain components in the program. This is what we have; we did not question ourselves if it is necessary for patients”.* Additionally, with that lack of evidence, it is hard to **predict dosage of PRP** and to decide when the **timing** is right **to finish** PRP: *“It is a difficult decision, at what point a patient is ready, and to reach consensus about this within the team and also with the patient….* ”.

### External factors

#### Shared factors

The majority of participants stated that financial factors should not influence dosage of PRP. Patients could imagine that **direct costs** that patients have to pay themselves, like travel costs, could be of concern for some patients, although they did not experience this problem themselves. (P11): *“It is regrettable if your own development depends on a few Euros extra per month”.* **Direct costs** of treatment and **indirect costs,** for example those related to returning to work, should not influence treatment because patients saw the costs of treatment as an **investment in the future**: (P9): *“Well, I think, on the longer term, if treatment works, you save money. It also applies for work: if my boss did not allow me time for this treatment, I might not yet be at work for 100%”.* **Personal and work factors** could have a positive or negative influence on the opportunity to receive treatment, and therefore could influence the satisfaction or choice for dosage of PRP. Some patients had to adjust their activities at home to their treatment: (P11): “*On treatment days, I did not have the energy to do things at home. I was happy I made the choice to delegate the household tasks. The days at home I had time to do things with my children and husband*”. A rehabilitation professional mentioned that sometimes, dosage of treatment can be adjusted to work or personal situations: *“If patients are not able to organize the required dosage of two times a week and they have strong reasons, we sometimes decide to reduce frequency of treatment to once a week”.*

#### Factors from patients only

Patients perceived **support from environment** (e.g. partner, colleagues, employer) as a positive factor because it can contribute to better results and may lower dosage, and thus helped with completing PRP successfully. Lack of support could negatively influence the results of the program and may lead to extension of PRP.

#### Factors from rehabilitation professionals only

In the focus groups, rehabilitation professionals mentioned that patient **travel time** could influence the choice of the offered **format of treatment,** therefore influencing the dosage of PRP. *“This center is a center of expertise regarding pain rehabilitation, so patients come from across the province, which makes outpatient rehabilitation not appropriate”.* If the patient received **injury compensation,** it sometimes can impede progression in functional improvements. Therefore, rehabilitation professionals stated that in some cases, this could be a reason for not starting PRP or for stopping it prematurely. **Test results or other treatments** can also influence the dosage of PRP: *“More often you see a sort of intermediate treatment if patients need other treatments, like EMDR ….. Or if test results are not available; waiting for these results causes delay*” (EMDR is the abbreviation for Eye Movement Desensitization and Reprocessing; trauma treatment).

### Differences and similarities

Interviews and focus groups were performed in three rehabilitation centers across the Netherlands. All centers offered multidisciplinary PRP for patients with CMP. Although factors were similar for all centers, dosages differed per center.. All patients, in general, mentioned being **satisfied** with the received dosage, although some had suggestions for improvements related to dosage of PRP. Rehabilitation professionals reported that they supported the choices for current dosages of PRP; however they also had suggestions for improvements and implications for further research in order to eliminate the lack of evidence for an optimal dosage of PRP.

## Discussion

As part of an ongoing exploration into factors related to optimizing dosage decisions of PRP, this study provides insight into perspectives of patients and rehabilitation professionals that in- or explicitely feed PRP dosage decisions. Factors important in relation to dosage of PRP were patient related characteristics, treatment related characteristics and external factors. In the patient related factors case complexity from a biopsychosocial perspective was important in determining dosage. Regarding treatment related characteristics, logistics played a role, including referral factors, the format of the program was considered and interactions between patients and professionals were taken into account. As external factors, support from others, costs, traveling distance and injury compensation were taken into account when deciding on dosage. Despite agreements in factors deemed important to establish dosage, the actual dosages differed to a large extent. For example, in general, patients need clarity about their dosage of the PRP at the start of program, but they would like to have the opportunity to adapt the dosage to their individual needs during PRP, in consultation with their rehabilitation professionals. Patients and rehabilitation professionals mentioned that personal characteristics, such as motivation and ability to change, general status of a patient, acceptance, and self-understanding, are taken into account when determining dosage. No explicit reasons for differences in choices of dosage were expressed during the focus groups, which suggests that PRP dosage choices are based on historical grounds, economics, routine and clinical expertise. This, in turn, can be explained from the absence of evidence to guide clinicians [5]. It seems that each rehabilitation center has its own ‘baseline-dose’, which forms a basis for choices to extend or shorten the PRP while taking into account factors mentioned in this study. These factors were similar between professionals of different centers.

Treatment outcome is the sum of specific and non-specific treatment effects. Specific effects are those effects that are unique for the treatment, for instance increase of grip strength after grip strengthening exercises, or behavioral modification after cognitive behavioural treatment. Non-specific effects are effects that are common for different types of treatments. These effects are related to for instance expectancy of results, a shared biopsychosocial approach in delivering PRP by team members, relationship factors like warmth, encouragement, listening but also to setting of the treatment. The factors mentioned in this study are linked to specific or non-specific effects. Content and format of treatment, and external factors (e.g. direct and indirect costs, investment in future, personal and work factors, support, test results) are factors that are taken into account when deciding for a certain dosage, and influence satisfaction with dosage. These factors could explain part of the differences in choices of the offered PRP dosage. However, the results of this study showed that these factors are not sufficient to explain the difference in dosage between centers.

It is hypothesized that in the absence of evidence regarding dosage of PRP, as mentioned in the focus groups, all rehabilitation centers made different choices regarding dosage. Current choices are in part based on content and dosage at the time of developing the PRP. Over time, dosage was adapted because of changes in society, costs, experiences, and new insights related to content or format of treatment. At present, however, no published evidence can support or refute the choices of dosage and therefore, rehabilitation professionals are not able to rationally choose optimum dosage of PRP.

### Strengths and limitations

Analyzing factors associated with dosage using a qualitative method from the perspective of patients and rehabilitation professionals is considered a strength of this study because it is the first study providing an overview of factors related to dosage from patient and professionals’ perspectives. Taking into account three leading PRPs with different dosages and similar content across the Netherlands is a strength because this provides insight into the differences and similarities regarding perspectives of dosage, resulting in similar factors regardless of dosage varieties. Although this study was performed in three centers, it focused solely on centers in the Netherlands, limiting generalization to PRPs performed in other countries. The qualitative nature of this study resulted in perspectives that cannot simply be generalized to all other groups of patients and rehabilitation professionals involved in PRPs. As this was a first and explorative study, many topics were covered and perspectives were raised during the interviews. While this methodology has lead to a broad range of factors that may be considered to decide on an optimum PRP dose, the downside is its lack of depth of these perspectives. Future studies should build on the results of this study to further explore these factors.

### Recommendations

This study is an initial exploration of the perspectives of patients and rehabilitation professionals regarding PRP dosage. It resulted in an overview of factors that can be used for future research and clinical practice regarding determining PRP dosage. Clinical practice might be improved by taking these factors into account when contemplating on dosage and making more explicit dosage decisions with a patient, being a mutual decision between professional and patient, geared towards overall success of PRP.

## Conclusions

In conclusion, this study shows that, although dosage of PRP differed, patients and rehabilitation professionals mentioned similar themes that influence dose choices: patient related characteristics (case complexity from a biopsychosocial perspective), treatment related characteristics (logistics and format of the program, interaction between patients and professionals), and external factors (support from others, costs, traveling distance and injury compensation). In absence of published evidence, differences in choices of PRP dosage appear mainly based on historical grounds and clinical expertise. Therefore, research is needed to guide choices of optimum PRP dosage.

## Additional file

Additional file 1: Semi-structured interview patients. Focus group interview rehabilitation professionals. (DOCX 16 kb)
