# Supplementary material for: Dosage of pain rehabilitation programs: a qualitative study from patient and professionals’ perspectives
Source: BMC Musculoskelet Disord. 2018 Jun 30;19:206. doi: 10.1186/s12891-018-2125-4 (PMC6026334; doi:10.1186/s12891-018-2125-4)
Supplement: Supplementary file 1 — Semi-structured interview patients. Focus group interview rehabilitation professionals. (DOCX 16 kb) [file 12891_2018_2125_MOESM1_ESM.docx]

**Appendices:**

**Semi-structured interview patients**

1. General questions about received treatment (type) and dosage (duration and intensity).
2. What did you think about received treatment, dosage of treatment and adaptations?

*Able to combine with other daily activities (work, hobbies, family life)?*

*To what extent did you have a voice in the dosage and adaptations?*

*When did treatment didn't have anything to add anymore?*

*To what extent are you satisfied about results?*

*Which factors contributed to the results?*

1. If you could design the treatment regarding dosage by yourself, what would it look like and which factors would influence your design?

*What would be the minimum amount of weeks?*

1. General questions about direct and indirect costs of the treatment and employment.

*How should these costs play a role in determining and adapting dosage of treatment?*

*How should costs for the employer play a role in determining dosage of the treatment?*

**Focus group interview rehabilitation professionals**

1. General questions about the present treatment (type) and dosage (duration and intensity).
2. How and when is dosage determined?

*What is the present program regarding dosage based on?*

*At what point and by whom is dosage determined?*

*Do patients and professionals always agree about the proposed duration?*

*Which* *factors are taken into account while determining or adapting dosage?*

*In what way is the patient involved?*

1. What do you think about the differences in dosage between pain rehabilitation centers?
2. What would the ideal multidisciplinary treatment look like?

*What would be the minimal dosage and why?*

*What would be the ideal dosage and why?*

*Which disciplines should be involved?*

*What factors should be taken into account while determining and adapting dosage?*

1. What can or should be changed about the dose of pain rehabilitation in the Netherlands or in general?
